# Acute Kidney Injury in the Intensive Care Unit: Recent Advances

**DOI:** 10.7759/cureus.109927

**Published:** 2026-05-30

**Authors:** Ibrahim Fadl Mahmoud, Saud M Erwi, Ali M Alfageeh

**Affiliations:** 1 Anesthesia, Intensive Care and Pain Management, Damietta Faculty of Medicine, Al-Azhar University, New Damietta, EGY; 2 Critical Care Unit, Armed Forces Hospital Jazan, Jazan, SAU; 3 Nephrology and Kidney Transplant Center, Armed Forces Hospital Southern Region, Khamis Mushait, SAU

**Keywords:** acute kidney injury, biomarkers, critical care nephrology, fluid stewardship, intensive care unit, renal replacement therapy, venous congestion

## Abstract

Acute kidney injury (AKI) in the intensive care unit (ICU) is a heterogeneous syndrome characterized by kidney stress, structural injury, functional decline, requirement for extracorporeal organ support, and incomplete recovery, rather than a single creatinine-defined event. Current critical care practice increasingly integrates conventional Kidney Disease: Improving Global Outcomes (KDIGO) diagnostic criteria with urine output trajectories, cystatin C measurement when serum creatinine is unreliable, structural damage biomarkers when results will change management, hemodynamic phenotyping, venous congestion assessment, nephrotoxin stewardship, fluid stewardship, and recovery planning. This narrative review summarizes recent advances relevant to adult ICU care, including evolving definitions of AKI and acute kidney disease, sepsis-associated microcirculatory and cellular injury, systemic venous congestion, point-of-care ultrasound-assisted congestion assessment, biomarker-triggered prevention bundles, individualized renal replacement therapy (RRT), continuous renal replacement therapy (CRRT), and post-ICU recovery pathways. Key recommendations include avoiding automatic fluid loading in response to oliguria; interpreting mean arterial pressure together with cardiac output, central venous pressure, right ventricular function, and intra-abdominal pressure; using biomarkers only within an actionable response pathway; and initiating RRT according to urgent or trajectory-based indications rather than serum creatinine alone. Precision ICU nephrology is best conceptualized as the timely alignment of phenotype, risk, trajectory, and organ support objectives.

## Introduction and background

Acute kidney injury (AKI) in the intensive care unit (ICU) is no longer adequately described as a secondary renal complication of critical illness. It is a central component of multiorgan dysfunction, a marker of illness severity, and an amplifier of pulmonary, cardiovascular, neurologic, metabolic, hematologic, and pharmacologic instability [1,2]. AKI affects approximately 30%-60% of critically ill adults, depending on the ICU population and diagnostic criteria. As a result, AKI is one of the most common forms of organ dysfunction encountered in intensive care medicine [2-4].

AKI affects a large proportion of critically ill adults, and severe AKI is associated with mortality, renal replacement therapy (RRT), prolonged mechanical ventilation (MV), longer ICU length of stay, incomplete kidney recovery, recurrent AKI, chronic kidney disease (CKD), cardiovascular events, frailty, readmission, and impaired quality of life [1-5].

Creatinine-centered diagnosis remains necessary but insufficient. Serum creatinine (SCr) rises late, is diluted by fluid accumulation, and is distorted by sarcopenia, liver dysfunction, catabolism, altered production, and changing volume of distribution. Urine output (UO) changes earlier but is non-specific and may reflect stress neurohormones, venous congestion, obstruction, low cardiac output (CO), vasoplegia, abdominal hypertension, or tubular injury. Modern ICU practice should therefore view AKI as a dynamic continuum that includes kidney susceptibility, kidney stress, subclinical injury, overt dysfunction, need for kidney support, acute kidney disease (AKD), and recovery or non-recovery [1-4,6,7].

These limitations show that traditional AKI paradigms relying solely on SCr and UO may not detect early kidney stress, structural injury, or reversible hemodynamic and congestive phenotypes. A modern ICU approach should integrate filtration markers, urine-output trends, clinical context, exposure assessment, perfusion, venous congestion, and recovery planning [1-4,6,7].

This review provides a bedside-oriented synthesis of recent advances in ICU AKI, with emphasis on definitions, pathobiology, phenotypes, diagnosis, biomarkers, kidney perfusion, venous congestion, fluid stewardship, nephrotoxicity, supportive care, RRT, and post-ICU kidney recovery. The goal is to provide a practical framework for individualized ICU kidney support and avoidance of potentially harmful interventions.

## Review

Review approach

This article presents a narrative, clinically focused review designed for adult critical care practice. The review methodology was explicitly defined. A structured literature search was conducted using PubMed/MEDLINE, Embase, Cochrane Central Register of Controlled Trials (CENTRAL), Scopus, Web of Science, Google Scholar, ClinicalTrials.gov, the World Health Organization International Clinical Trials Registry Platform, and relevant society or guideline websites, including Kidney Disease: Improving Global Outcomes (KDIGO) and Acute Disease Quality Initiative (ADQI) resources. The search encompassed publications from January 2012 to April 2026, with older landmark studies included when essential for definitions, physiological context, or major practice-changing evidence.

We used Boolean operators to combine search terms, which included AKI, AKD, critically ill, ICU, sepsis-associated AKI, septic shock, kidney biomarkers, cystatin C, NGAL, tissue inhibitor of metalloproteinases-2, insulin-like growth factor-binding protein 7, renal perfusion, venous congestion, venous excess ultrasound (VExUS), fluid stewardship, balanced crystalloids, nephrotoxicity, contrast-associated AKI, continuous kidney replacement therapy, continuous RRT (CRRT), RRT timing, regional citrate anticoagulation, renal recovery, and post-AKI follow-up.

We prioritized international guidelines, consensus statements, randomized controlled trials, systematic reviews, meta-analyses, large observational cohorts, and physiology-based reviews relevant to adult ICU management. We excluded pediatric studies, isolated case reports, non-English reports when reliable English data were available, studies of non-critical care populations without ICU applicability, and studies superseded by higher-quality evidence. This article provides a narrative, clinically oriented review intended for adult critical care practice. As it is not a formal systematic review, PRISMA flow diagrams, protocol registration, duplicate screening, formal risk-of-bias assessment, and pooled meta-analysis were not conducted. Evidence was synthesized based on bedside relevance, mechanistic plausibility, study design, alignment with contemporary guidelines and consensus statements, and implications for ICU decision-making.

Definitions and conceptual framework

The KDIGO criteria remain the primary standard for diagnosing AKI: an increase in SCr of at least 0.3 mg/dL within 48 hours, an increase to at least 1.5 times baseline within seven days, or UO of less than 0.5 mL/kg/hour for at least six hours [1].

The KDIGO 2026 public review draft expands the existing framework by emphasizing structural kidney abnormalities, the selective use of cystatin C, regulatory-qualified damage biomarkers, distinctions between persistent and transient AKI, AKD, and recovery status. Until the final publication, these statements should be regarded as evolving draft guidance rather than definitive recommendations [8].

Several distinctions are clinically significant. Functional AKI reflects reduced filtration from reversible hemodynamic, neurohormonal, or pressure-related mechanisms, whereas structural AKI involves tubular, endothelial, interstitial, or glomerular injury. These patterns often overlap. Septic shock, for example, may combine vasoplegia, preserved or increased renal blood flow, microvascular shunting, tubular stress, mitochondrial dysfunction, and venous congestion [3,9,10]. Table 1 defines and categorizes AKI in critical illness.

**Table 1:** Definitions and conceptual categories of AKI in critical illness AKI, acute kidney injury.

Category	Conventional definition	Modern interpretation	Bedside implication	Source references
Acute kidney injury	Kidney Disease: Improving Global Outcomes (KDIGO): serum creatinine and/or urine output change	Functional syndrome of stress, injury, dysfunction, and failed adaptation	Do not assume uniform biology or reversibility	[1-4,8]
Subclinical acute kidney injury	Damage biomarker-positive without diagnostic serum creatinine or urine output threshold	Structural injury or kidney stress before overt excretory decline	Window for prevention, monitoring, and exposure minimization	[6,7]
Transient acute kidney injury	Rapid reversal, usually within 48 hours	Often hemodynamic or adaptive but not always benign	Reassess perfusion, congestion, and drug burden	[1,4,8]
Persistent acute kidney injury	Ongoing dysfunction beyond 48 hours and up to seven days	Greater structural injury and higher acute kidney disease risk	Closer surveillance and lower threshold for nephrology input	[4,5,8,11,12]
Acute kidney disease	Kidney abnormalities lasting 7 to 90 days	Bridge from acute kidney injury to chronic kidney disease with active biology	Plan follow-up and kidney protection after discharge	[4,5,8,11,12]

The progression from AKI to AKD and subsequently to CKD should be explicitly documented. Kidney abnormalities persisting beyond 7 days and up to 90 days define AKD, a period characterized by incomplete repair, ongoing inflammation, fibrosis, capillary rarefaction, hypertension, albuminuria, and residual drug exposure vulnerability. ICU discharge summaries should therefore include AKI stage, probable phenotype, RRT exposure, unresolved kidney dysfunction, nephrotoxin events, and follow-up plan [4,5,11,12]. Table 2 presents the AKI severity staging system.

**Table 2:** Severity staging system for AKI AKI, Acute kidney injury; C1-C3, serum creatinine severity stages; U1-U3, urine output severity stages; B0/B1, biomarker-negative/biomarker-positive status.

Domain	Stage	Criteria	Interpretation	Source references
Serum creatinine	C1	Increase of 0.3 mg/dL (26.5 micromol/L) or 1.5-1.9 times baseline	Mild functional criterion, requiring clinical context and trajectory assessment	[1,8]
Serum creatinine	C2	2.0-2.9 times baseline	Moderate functional criterion associated with a higher risk	[1,8]
Serum creatinine	C3	3.0 times baseline, increase to 4.0 mg/dL (353.6 micromol/L), or renal replacement therapy initiation	Severe functional criterion or kidney support requirement	[1,8]
Urine output	U1	Less than 0.5 mL/kg/hour for 6 to 12 hours	Early urine output criterion; evaluate phenotype before intervention	[1,8]
Urine output	U2	Less than 0.5 mL/kg/hour for more than 12 hours	Persistent oliguria with increased risk	[1,8]
Urine output	U3	Less than 0.3 mL/kg/hour for more than 24 hours or anuria for more than 12 hours	Severe urine output criterion; urgent reassessment required	[1,8]
Damage biomarker	B0/B1	Absence or presence of elevated damage biomarker	Structural injury or stress may be identified before creatinine rises	[6-8]

Pathophysiology: from renal blood flow to cellular adaptation

Renal autoregulation normally buffers changes in perfusion pressure through myogenic tone, tubuloglomerular feedback, afferent and efferent arteriolar responses, and neurohormonal signaling. Critical illness disrupts this system through vasoplegia, inflammation, endothelial dysfunction, catecholamine stress, MV, altered intrathoracic pressure, abdominal hypertension, and venous congestion [2,3,9,13,14]. A conventional mean arterial pressure (MAP) may not ensure adequate kidney oxygenation if the renal microcirculation is maldistributed, venous pressure is high, or tubular oxygen demand exceeds supply.

Sepsis-associated AKI illustrates this shift in thinking. Older models emphasized global renal ischemia and acute tubular necrosis. Contemporary translational and clinical data suggest a multifactorial syndrome involving endothelial injury, glycocalyx disruption, leukocyte adhesion, intrarenal shunting, capillary leak, inflammatory signaling, mitochondrial dysfunction, and maladaptive cell-cycle arrest [3,9,10].

Microvascular shunting involves redistribution of renal blood flow, resulting in a mismatch between perfusion and oxygen utilization [9]. Maladaptive cell-cycle arrest is a persistent injury-associated cellular response that is linked with impaired recovery. These mechanisms can coexist with apparently adequate systemic blood pressure. This emphasizes that systemic hemodynamics alone do not fully describe kidney oxygenation or tubular stress.

Venous congestion represents a significant and often under-recognized contributor to kidney dysfunction. Venous congestion refers to elevated venous pressure that impairs renal drainage and filtration [10,13-15]. Factors such as elevated central venous pressure (CVP), right ventricular (RV) dysfunction, pulmonary hypertension, portal or hepatic venous pulsatility, renal venous Doppler abnormalities, fluid overload, and intra-abdominal hypertension diminish the transrenal perfusion gradient and elevate renal interstitial pressure.

In these patients, additional fluid administration may further impair filtration despite transient improvements in stroke volume. MV may exacerbate this process by increasing intrathoracic pressure, reducing venous return, increasing RV afterload, and elevating renal venous pressure. These observations underscore that kidney perfusion is determined by inflow, outflow, parenchymal constraint, and cellular oxygen consumption, rather than MAP alone [10,13-16].

ICU phenotypes and risk stratification

Phenotyping helps convert AKI from a descriptive label into a management strategy. The low-flow phenotype is dominated by reduced CO or arterial underfilling and may require restoration of effective perfusion. The septic-inflammatory phenotype is characterized by vasoplegia, endothelial injury, inflammatory signaling, microvascular mismatch, and immune-metabolic stress; treatment focuses on source control, vasopressor optimization, cautious fluids, and avoidance of unnecessary nephrotoxins [2,3,9,10]. The congestive phenotype is dominated by high CVP, RV dysfunction, portal or hepatic venous pulsatility, fluid overload, and impaired renal outflow; it usually requires decongestion rather than repeated boluses [13-15].

Other phenotypes remain essential. Nephrotoxic AKI reflects exposure to aminoglycosides, vancomycin, amphotericin, calcineurin inhibitors, iodinated contrast, non-steroidal anti-inflammatory drugs, antivirals, chemotherapies, or immune checkpoint inhibitors. Postoperative AKI combines ischemia-reperfusion, inflammation, hemodilution, hemolysis, vasoplegia, hypotension, fluid accumulation, and drug exposure [16-18]. Obstructive AKI should be rapidly excluded in abrupt oliguria or anuria. Most ICU patients have mixed phenotypes, and iterative reassessment is more useful than forcing a single diagnosis.

Risk stratification should combine susceptibility and exposure. Susceptibility includes CKD, albuminuria, diabetes, heart failure, cirrhosis, advanced age, frailty, sarcopenia, malignancy, previous AKI, and reduced renal reserve. Exposure includes sepsis, shock, vasopressors, major surgery, nephrotoxins, contrast, MV, positive fluid balance, rhabdomyolysis, and abdominal hypertension. High-risk patients need active prevention, not passive creatinine surveillance [8]. Table 3 summarizes major ICU AKI phenotypes, underlying mechanisms, hemodynamic profiles, and bedside implications.

**Table 3:** Practical intensive care unit acute kidney injury phenotypes and immediate bedside implications

Phenotype	Dominant mechanism	Clinical clue	Immediate priority	Source references
Low-flow	Reduced forward flow or arterial underfilling	Low cardiac output, shock, and rising lactate	Restore effective perfusion; avoid blind overfilling	[2,3,9]
Septic-inflammatory	Endothelial injury, vasoplegia, cellular stress, and microvascular mismatch	Sepsis, vasopressors, and variable renal blood flow	Source control, vasopressor-supported perfusion, cautious fluids, and nephrotoxin avoidance	[3,9,10]
Congestive	High renal venous pressure and renal interstitial pressure	Right ventricular dysfunction, high central venous pressure, edema, and venous Doppler abnormalities	Decongest and optimize right ventricular function; avoid reflex fluid boluses	[10,13-15]
Nephrotoxic	Tubular, interstitial, crystal, hemodynamic, or immune-mediated injury	Recent drug or contrast exposure	Stop, substitute, monitor drug levels, and adjust doses	[2,3]
Postoperative	Inflammation, ischemia-reperfusion, hemodilution, vasoplegia, and exposures	Major surgery, cardiopulmonary bypass, hypotension, and biomarker positivity	Prevention bundle and surveillance	[19,20]
Obstructive	Postrenal pressure transmission	Abrupt oliguria or anuria, hydronephrosis, and catheter obstruction	Ultrasound and urgent relief of obstruction	[1,8]

Diagnosis, biomarkers, and precision recognition

Diagnosis begins with SCr and UO, but results must be interpreted in context. SCr may underestimate injury in fluid-overloaded or sarcopenic ICU patients. Oliguria may reflect adaptive sodium and water retention, venous congestion, obstruction, or tubular stress. Urinalysis and sediment remain valuable, low-cost tools: pigment casts suggest rhabdomyolysis; dysmorphic hematuria and proteinuria suggest glomerular disease; pyuria may indicate interstitial nephritis; and granular casts support tubular injury. Fractional excretion indices can be misleading in sepsis, CKD, cirrhosis, diuretic exposure, contrast exposure, and mixed phenotypes [2,3].

Biomarkers expand diagnosis beyond filtration assessment. Neutrophil gelatinase-associated lipocalin (NGAL) rises early after tubular stress but may be confounded by inflammation or infection. Kidney injury molecule-1 (KIM-1) is more specific to proximal tubular injury but is less routinely available. Interleukin-18 (IL-18) and liver-type fatty acid-binding protein (L-FABP) reflect inflammatory or oxidative pathways. Tissue inhibitor of metalloproteinases-2 (TIMP-2) and insulin-like growth factor-binding protein 7 (IGFBP7) reflect cell-cycle arrest and may identify kidney stress before SCr rises [6,16-18].

Cystatin C and proenkephalin are biomarkers that primarily reflect renal function. Cystatin C is less influenced by muscle mass than creatinine and may enhance the detection of AKI in patients with sarcopenia or fluid overload, although its interpretation can be affected by inflammation and corticosteroid use. Proenkephalin has emerged as a promising dynamic filtration marker in sepsis and critical illness, with potential to detect changes earlier than creatinine in certain contexts; however, its routine clinical implementation remains limited [6,7,16].

The key issue is not whether a biomarker predicts AKI, but whether it informs management. Evidence is strongest for biomarker-guided prevention bundles in high-risk perioperative patients, especially after cardiac and major surgery. Biomarker results should be interpreted according to the clinical context and should be used only when they trigger an actionable pathway, such as intensified monitoring, nephrotoxin avoidance, hemodynamic optimization, medication review, and avoidance of unnecessary hyperchloremia or fluid accumulation [6-8,19,20]. Indiscriminate testing without a predefined response pathway may increase cost and uncertainty without improving patient-centered outcomes.

Hemodynamics, congestion, and fluid stewardship

Effective kidney protection depends on the integration of arterial pressure, CO, vascular tone, CVP, intra-abdominal pressure, intrathoracic pressure, and fluid balance. While the MAP target of 65 mmHg is appropriate for many patients, those with chronic hypertension or impaired autoregulation may require individualized targets. Increasing MAP without concurrent improvement in CO or relief of venous congestion may not enhance kidney function. Mean perfusion pressure, calculated as MAP minus CVP, is an imperfect but clinically useful metric because it emphasizes venous pressure as renal afterload rather than solely a preload estimate [10,13,15].

Point-of-care ultrasound and venous Doppler imaging can refine phenotyping. Assessment of left ventricular function, RV function, pericardial constraint, valvular disease, inferior vena cava dimensions, hepatic vein flow, portal venous pulsatility, and intrarenal venous Doppler patterns may identify systemic venous congestion. VExUS has increased bedside recognition of systemic venous congestion, although current supporting evidence remains predominantly observational and should not be interpreted as proof that VExUS-guided therapy improves outcomes [14,15]. Its value is greatest when integrated with perfusion, oxygenation, vasopressor requirements, capillary leak, cumulative fluid balance, and clinical trajectory.

Intravenous fluids require a prescription with a clear indication, specified dose, defined reassessment interval, monitoring for adverse effects, and explicit discontinuation criteria. Fluid resuscitation may be necessary during early shock with arterial underfilling, but subsequent accumulation can exacerbate renal venous pressure, interstitial edema, abdominal pressure, and pulmonary mechanics. Balanced crystalloids are generally preferred in many ICU patients because chloride-rich saline may induce hyperchloremic acidosis and renal vasoconstriction; however, clinical trials show modest and context-dependent renal benefits rather than universal protection [21-23].

Fluid responsiveness should be distinguished from fluid tolerance. Although a patient may demonstrate increased stroke volume following a fluid bolus, this response can still result in harm due to exacerbation of pulmonary edema, abdominal hypertension, or venous congestion. Oliguria should prompt a comprehensive phenotype assessment rather than automatic fluid administration. After shock is controlled, de-resuscitation with diuretics or ultrafiltration should be considered in patients with fluid overload, particularly when congestion contributes to AKI [2,3,13-15].

Nephrotoxicity and medication stewardship

Drug-associated AKI in the ICU is usually multifactorial. Risk depends on the cumulative nephrotoxic burden, hemodynamic instability, inflammation, renal reserve, and extracorporeal clearance, rather than on a single agent. Aminoglycosides, vancomycin, amphotericin B, calcineurin inhibitors, non-steroidal anti-inflammatory drugs, iodinated contrast, antivirals, chemotherapies, proton-pump inhibitors, beta-lactams, and immune checkpoint inhibitors may cause tubular toxicity, altered intraglomerular hemodynamics, crystal nephropathy, acute interstitial nephritis, thrombotic microangiopathy, or pseudo-AKI [2,3,24].

Effective stewardship starts with exposure mapping. For each patient with evolving AKI, conduct a systematic medication review to identify nephrotoxic agents, drugs needing renal dose adjustment, drugs requiring therapeutic drug monitoring, and medications that can be discontinued. When possible, area under the curve-guided vancomycin dosing should be used to reduce excessive exposure compared with trough-only strategies. For patients receiving CRRT, antibiotic dosing should account for effluent rate, residual kidney function, volume of distribution, protein binding, membrane characteristics, downtime, and infection severity [25].

Contrast-associated AKI requires careful interpretation. Risk is often overestimated when sepsis, hypotension, CKD, and concurrent nephrotoxins are not separated from contrast exposure. Clinically indicated imaging should proceed when it is likely to change management. Prevention should focus on avoiding unnecessary repeat exposure, minimizing contrast dose, optimizing hemodynamics, avoiding concurrent nephrotoxins when possible, and providing isotonic crystalloid hydration to high-risk patients when appropriate [8].

Supportive management of established AKI

No pharmacologic therapy reliably reverses established ICU AKI in diverse patient populations. Supportive care is the primary treatment. Key priorities are source control, relieving obstruction, optimizing hemodynamics, decongestion when indicated, correcting abdominal hypertension, treating rhabdomyolysis, discontinuing nephrotoxins, adjusting renal dosing, and preventing avoidable complications [1-3,8]. The first 24 to 72 hours after diagnosis are critical, as preventable exposures and fluid accumulation can worsen injury. Monitoring should include UO trends, cumulative fluid balance, electrolytes, acid-base status, lactate, hemodynamic profile, vasopressor requirements, ventilation settings, nutrition, and relevant drug levels [1-3,8].

Hyperkalemia requires immediate membrane stabilization, intracellular potassium shift, removal from the body, and addressing the underlying cause. Severe fluid overload with pulmonary edema or hypoxemia should prompt decongestion or RRT. Uremic complications such as encephalopathy, pericarditis, bleeding, or severe catabolic burden require urgent escalation of care [1,19-21].

Metabolic acidosis should be assessed based on underlying mechanisms. Sodium bicarbonate is not indicated for all cases of low bicarbonate but may be appropriate in severe acidemia, especially with moderate-to-severe AKI and adequate respiratory reserve. The BICAR-ICU showed reduced RRT use and possible benefit in the AKI subgroup, while BICARICU-2 did not demonstrate an overall mortality benefit. Bicarbonate therapy should be tailored to pH severity, hemodynamic impact, ventilatory capacity, sodium load, calcium effects, and the timing of definitive therapy [26,27].

Nutrition and pharmacology are essential components of kidney support. Catabolic ICU patients with AKI require sufficient protein, and needs may increase during RRT due to losses of amino acids, vitamins, trace elements, and drugs through the extracorporeal circuit. Anemia and transfusion decisions should be based on critical care indications, not AKI alone, while recognizing that severe uremia can impair platelet function [1-3].

Supportive care should be tailored to the specific AKI phenotype. In septic AKI, kidney protection relies on rapid infection control, appropriate antimicrobial administration, vasopressor-supported perfusion, avoidance of unnecessary chloride and fluid excess, and awareness that creatinine improvement may lag behind systemic recovery. In congestive AKI, management should prioritize RV function, venous pressure reduction, deresuscitation, and optimization of respiratory mechanics; additional fluid boluses are often detrimental. In postoperative AKI, preventive measures should be implemented before creatinine elevation, particularly in high-risk patients identified by comorbidities, surgical stress, hemodynamic instability, or biomarkers. In cases of suspected immune-mediated, glomerular, or interstitial disease, diagnostic delays can threaten kidney function, and nephrology consultation should be sought promptly, without waiting for dialysis criteria [2,3,14,17].

RRT: timing, modality, delivery, and discontinuation

RRT should be viewed as extracorporeal organ support rather than dialysis for a creatinine number. Urgent indications include refractory severe hyperkalemia, severe metabolic acidosis refractory to medical therapy, diuretic-resistant pulmonary edema or severe hypoxemia due to volume overload, uremic complications, severe intoxication with a dialyzable toxin, and selected cases of severe hyperammonemia. Relative indications include persistent severe AKI, worsening non-renal organ dysfunction due to fluid or solute accumulation, high anticipated solute burden, limited physiologic reserve, and inability to provide nutrition because of fluid restriction [1,8].

Large trials have changed the timing debate. Although the Early Versus Late Initiation of RRT in Critically Ill Patients with Acute Kidney Injury (ELAIN) trial suggested benefit from earlier RRT initiation in a selected single-center high-risk population, larger multicenter trials, including AKIKI, IDEAL-ICU, STARRT-AKI, and AKIKI-2, did not demonstrate a consistent survival benefit from accelerated RRT initiation in patients without urgent indications [28-32]. A practical approach is therefore safe deferral with close monitoring, predefined escalation triggers, and avoidance of both unnecessary extracorporeal therapy and dangerous delay.

RRT modality should match physiology. Intermittent hemodialysis provides rapid solute and potassium removal and is appropriate for hemodynamically stable or recovering patients. CRRT is preferred in vasopressor-dependent shock, severe fluid overload, acute brain injury, advanced liver failure, major sodium or osmolality concerns, and situations requiring precise ultrafiltration. Prolonged intermittent RRT or sustained low-efficiency dialysis is a hybrid option for intermediate instability. Acute peritoneal dialysis may be useful when vascular access is difficult, anticoagulation is contraindicated, extracorporeal platforms are unavailable, or resources are limited, but clearance and ultrafiltration are less predictable [33]. Table 4 summarizes RRT modality selection in adult ICU AKI.

**Table 4:** Renal replacement therapy modality selection in adult intensive care unit acute kidney injury

Modality	Best fit	Main advantage	Main caution	Source references
Intermittent hemodialysis	Hemodynamically stable patient needing rapid solute, potassium, toxin, or acid-base correction	Fast correction and broad availability	Hypotension, osmotic shifts, and limited fluid precision	[1,8,33]
Continuous kidney replacement therapy or continuous renal replacement therapy	Vasopressor-dependent shock, severe congestion, acute brain injury, liver failure, or need for precise ultrafiltration	Continuous solute and fluid control with better hemodynamic tolerability	Downtime, anticoagulation, drug-dosing complexity, and resource intensity	[1,33,34]
Prolonged intermittent renal replacement therapy or sustained low-efficiency dialysis	Intermediate instability or transition from continuous therapy	Operational flexibility with gentler clearance than conventional intermittent hemodialysis	Less continuously adjustable than continuous therapy	[1,33]
Acute peritoneal dialysis	Limited vascular access, bleeding risk, or resource-constrained settings	No extracorporeal circuit and no routine systemic anticoagulation	Slower clearance and limitations from abdominal pathology or ventilation mechanics	[1,8,33]

CRRT modality selection involves choosing among diffusive, convective, mixed diffusive-convective, or ultrafiltration-only methods. The main subtypes are continuous venovenous hemodialysis (CVVHD), continuous venovenous hemofiltration (CVVH), continuous venovenous hemodiafiltration (CVVHDF), and slow continuous ultrafiltration (SCUF). Selection should match the primary clinical objective: CVVHD for steady small solute control, CVVH for convective clearance, CVVHDF for comprehensive solute and fluid management, and SCUF for controlled decongestion with minimal metabolic impact. These modalities are usually delivered continuously over 24 hours, with blood flow rates of 100-250 mL/min [1,2,29-33].

Current practice targets a delivered effluent rate of about 20-25 mL/kg/h for most patients, as higher intensities have not improved mortality in major trials [1,29-33]. Ultrafiltration rates should be adjusted dynamically. The optimal net fluid removal rate depends on factors such as vascular refill, vasopressor needs, venous congestion, RV function, capillary leak, and the patient’s overall organ support goals [29-32]. Regional citrate anticoagulation can prolong circuit life and reduce bleeding compared to systemic heparin but requires monitoring of calcium, acid-base status, total to ionized calcium ratio, and citrate accumulation risk in shock or severe liver dysfunction. When citrate is unsuitable or unavailable, unfractionated heparin remains a reasonable alternative, although it increases bleeding risk and may reduce circuit longevity in certain settings [34].

Discontinuation should be as deliberate as initiation. Trial off RRT is reasonable when native kidney function can maintain potassium, acid-base balance, solute control, and fluid balance without extracorporeal support. Rising UO, improving hemodynamics, stable electrolytes, falling catabolic burden, and achievable fluid goals support liberation. Creatinine during RRT should not be used alone to judge recovery [35].

Special ICU contexts

In acute respiratory distress syndrome, restrictive fluid management after shock resolution can improve lung mechanics and reduce venous pressure. However, excessive fluid removal may lower CO and impair kidney perfusion. In RV failure, the kidney faces venous hypertension, reduced forward flow, and elevated intrathoracic pressures. Management should focus on reducing RV afterload, optimizing oxygenation and ventilation, selecting appropriate vasopressors, and careful decongestion. In cirrhosis, AKI may result from hypovolemia, infection, hepatorenal physiology, bile cast nephropathy, abdominal hypertension, or nephrotoxicity. Treatment should include albumin, vasoconstrictors, infection control, and avoidance of nephrotoxins when hepatorenal physiology is suspected [8].

Prevention of postoperative AKI is preferable to reactive management. High-risk patients should be protected from sustained hypotension, excessive chloride administration, unnecessary nephrotoxins, severe anemia, hyperglycemia, and occult fluid accumulation. Biomarker-positive patients after major surgery should not be regarded as inevitably progressing to kidney failure; rather, they represent an actionable risk period during which adherence to care bundles can reduce moderate or severe AKI [19,20]. In trauma and burn patients, factors such as rhabdomyolysis, pigment nephropathy, compartment syndrome, shock, and extensive resuscitation may interact. Among oncology intensive care patients, checkpoint inhibitor nephritis, tumor lysis syndrome, obstructive uropathy, sepsis, and nephrotoxic antimicrobials frequently coexist.

Recovery, AKD, and long-term outcomes

Renal recovery after ICU AKI is not binary. Patients may have complete recovery, partial recovery, dialysis independence with reduced reserve, persistent AKD, recurrent AKI, albuminuria, hypertension, or progression to CKD. Risk increases with AKI severity, duration, RRT exposure, pre-existing CKD, older age, diabetes, cardiovascular disease, incomplete recovery at discharge, and recurrent nephrotoxin exposure [4-5,11-12]. Even apparently resolved AKI is associated with long-term kidney and cardiovascular risk.

**Figure 1:**
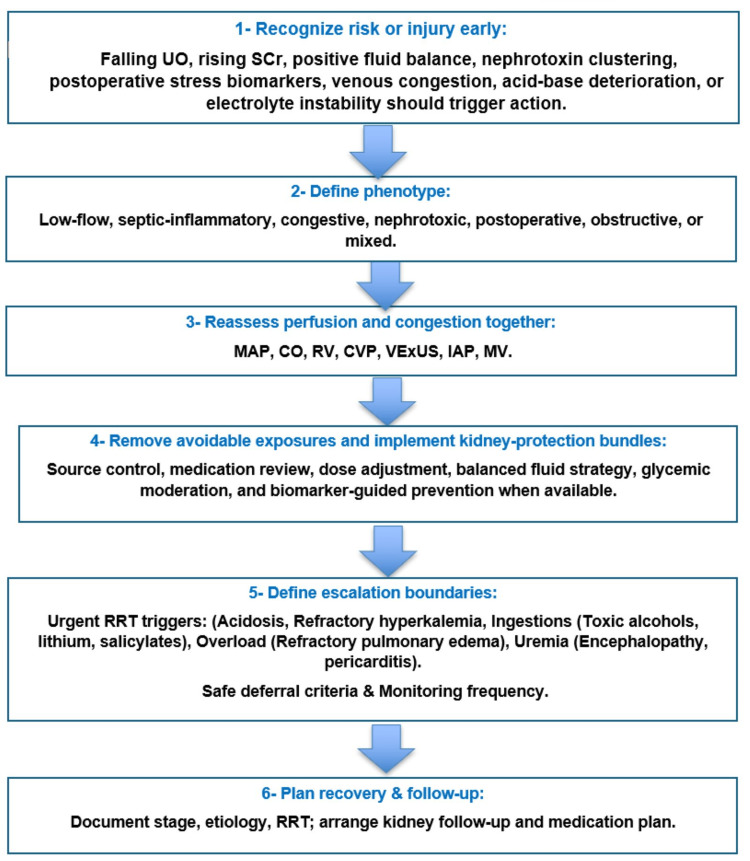
Clinical algorithm for acute kidney injury management in the intensive care unit The pathway emphasizes recognition, phenotyping, perfusion-congestion assessment, exposure removal, renal replacement therapy trigger definition, liberation planning, recovery, and follow-up. CO, cardiac output; CVP, central venous pressure; IAP, intra-abdominal pressure; MAP, mean arterial pressure; MV, mechanical ventilation; RV, right ventricle; RRT, renal replacement therapy; SCr, serum creatinine; UO, urine output; VExUS, venous excess ultrasound. Source references: [1-8,10-15,19,20,28-35]

Discharge planning is an important part of ICU AKI care. The discharge summary needs to include the highest AKI stage, presumed phenotype, any RRT exposure, unresolved kidney problems, nephrotoxin events, medication changes, and a follow-up plan [4,5,11,12]. Patients who have stage 3 AKI, need dialysis, did not fully recover, have CKD, significant albuminuria, repeated AKI, or an unclear diagnosis should get early follow-up with nephrology. Blood pressure, creatinine, cystatin C, where useful, urine albumin-to-creatinine ratio, electrolytes, medication safety, and renin-angiotensin system inhibitor reintroduction should be reviewed within a structured post-AKI pathway [11,12]. Figure 1 summarizes the proposed clinical algorithm for AKI management in the ICU.

## Conclusions

AKI in the ICU represents a heterogeneous syndrome characterized by kidney stress, structural injury, functional decline, need for organ support, and risk of impaired recovery. Recent advances have shifted clinical practice from delayed recognition based on creatinine levels to an integrated assessment that includes susceptibility, exposure, UO trajectory, biomarkers, hemodynamic phenotype, venous congestion, fluid tolerance, nephrotoxic burden, and long-term recovery. A key clinical challenge is to differentiate patients requiring increased perfusion from those needing decongestion, those suitable for watchful waiting from those requiring urgent RRT, and those with improved creatinine from those with ongoing kidney risk.

The most significant advances are both conceptual and operational. These include treating fluids as pharmacologic agents, considering venous pressure as renal afterload, using biomarkers only when they inform preventive strategies, individualizing RRT, and planning for kidney recovery before ICU discharge. Future progress will rely on integrating digital surveillance, mechanistic phenotyping, biomarker-guided pathways, and high-quality supportive care into standardized ICU systems.
